# Immunological considerations of modern animal models of malignant primary brain tumors

**DOI:** 10.1186/1479-5876-7-84

**Published:** 2009-10-08

**Authors:** Michael E Sughrue, Isaac Yang, Ari J Kane, Martin J Rutkowski, Shanna Fang, C David James, Andrew T Parsa

**Affiliations:** 1 Department of Neurological Surgery, University of California at San Francisco, San Francisco, California, USA

## Abstract

Recent advances in animal models of glioma have facilitated a better understanding of biological mechanisms underlying gliomagenesis and glioma progression. The limitations of existing therapy, including surgery, chemotherapy, and radiotherapy, have prompted numerous investigators to search for new therapeutic approaches to improve quantity and quality of survival from these aggressive lesions. One of these approaches involves triggering a tumor specific immune response. However, a difficulty in this approach is the the scarcity of animal models of primary CNS neoplasms which faithfully recapitulate these tumors and their interaction with the host's immune system. In this article, we review the existing methods utilized to date for modeling gliomas in rodents, with a focus on the known as well as potential immunological aspects of these models. As this review demonstrates, many of these models have inherent immune system limitations, and the impact of these limitations on studies on the influence of pre-clinical therapeutics testing warrants further attention.

## The Potential Promise of Immunotherapy for Primary Brain Tumors

Primary central nervous system (CNS) malignancies, though of low incidence in relation to many adult solid tumors, represent a disproportionately large fraction of cancer deaths due to their highly aggressive and fatal character. For example, Glioblastoma Multiforme (GBM), the most common and malignant brain tumor of adults, carries a median survival of less than 1 year. While current approaches to brain tumor therapy, including surgical resection, radiotherapy, and either systemic or local chemotherapy with either nitrosoureas or temozolamide, appear to prolong survival for patients with CNS cancers, the modest effect of these therapies, and their associated morbidity, has left investigators in search of alternative and novel treatments to extend quantity and quality of life for affected patients [1].

The nearly infinite flexibility and remarkable cellular specificity of the human immune response makes immune based approaches an attractive option to current therapy, which either crudely target entire regions of the brain (e.g. surgery, radiation), or potentially interfere with the cellular metabolism of all dividing cells in the body (e.g. alkylating agents). However, immunotherapy is not without technical barriers, which have hindered its incorporation into the therapeutic arsenal for treating CNS tumors. One such barrier is the known paucity of surface antigens unique to glioma cells, against which an immune response could be mounted. Another is the significant degree of local and systemic immunosuppression known to occur in glioma patients.

Perhaps the most significant hurdle to translating immunotherapeutic concepts into effective treatments for primary brain tumor patients is the fact that animals generally do not spontaneously develop CNS neoplasms, and, consequently, pre-clinical studies rely on artificial systems for basing conclusions regarding approaches being considered for use in patients. It is crucial that tumors artificially created in animal hosts for the purpose of developing immune based therapies, faithfully recapitulate the antigenic and immunological reality that exists in brain tumor patients. Artefactual inaccuracies could falsely suggest the efficacy of ineffective treatments [2], or worse, lead investigators to disregard effective ones. Given the limitations of the existing artificial systems used in pre-clinical studies, a critical evaluation of immunological considerations associated with the approaches used to create brain tumors in animals is essential prior to using these models to evaluate immune based therapies.

## Observed and Anticipated Immunological Deficiencies in Various Brain Tumor Models

While there exist a multitude of methods for introducing glial-type neoplasms into the rodent CNS, which histologically mimic human primary tumors, these methods can be described as belonging to one of two groups: 1) Tumors created by methods which do not target a specific gene, and 2) Tumors created by targeted mutation of genes known to be mutated in human tumors (i.e. gene specific methods) [3].

### Non-Specific Methods

It has been known since the 1970's that repetitive intravenous administration of nitrosourea compounds such as methynitrosourea (MNU) and N-ethyl-N-nitrosourea (ENU) produces glial-type neoplasms in immunocompetent rats [4]. However, the long time required to induce neoplasms, and inconsistency of tumor development, led to a shift towards implantation of neoplastic cells propagated in vitro [4].

While the majority of these models involve the use of rodent glioma cells injected in syngeneic hosts, it is also possible to use human glioma cells *in vivo *via their implantation in athymic mice. The pan-immune alterations seen in these rodents obviously limits the use of the xenograft models in some immunologic investigations, namely studies involving T-cell related immunity. These models however do maintain some aspects of their native immune systems and thus can be used to study some aspects of innate immunity [5], cytokine function [6], and natural killer cell function [7].

While rodent tumor cells implanted in rodent hosts have been widely used to study the interaction of brain tumors and the immune system, a number of major concerns with this approach have been reported. The first is these methods' dependence on cell culture for the production of neoplastic cells to implant. For example, we have shown that glioma cells long removed from their native histological milleu are immunologically different than similar cells immediately *ex vivo*, including changes in MHC and FasL expression and cytokine production; changes which apparently begin as soon as the first passage *in vitro *[8]. Consistent with these observations, expression profiling of patient tumors vs. corresponding cell cultures have revealed widespread changes gene expression once a tumor is subjected to in vitro growth conditions [9].

As well, while many of these models involve implantation of cells into animals derived from the cell-line originating strain, these cells still represent a graft, and unfortunately too often behave immunologically like foreign cells. Most syngeneic graft based models of brain tumors have been shown to induce an immunological response against implanted tumor cells [4]. For example, one of the original implantation models, the 9L Gliosarcoma model, was initially created in Fischer rats using serial MNU injections [10], and has been widely used to evaluate various immunotherapic therapies [11-15]. However, investigations have demonstrated the 9L model is relatively immunogenic, and that it is possible to immunize animals against these tumors using irradiated 9L cells, implying that they are viewed as foreign tissue [16]. We have demonstrated the occurrence of a similar phenomenon in the C6 glioma cell line, as rats subjected to simultaneous intracerebral and subcutaneous glioma cell implantation experienced a nearly 9 fold improvement in survival compared to those subjected to intracerebral implantation alone [2]. As well the 9L Fischer model has been demonstrated to induce a similar immune response. Other models such as CNS-1 cell implantation in Lewis rats have been found to induce less of an immune response [4]. Thus, variability in immune response occurs in a number of these models, and this should be taken into consideration when evaluating immunotherapies in these models.

There are significantly fewer syngeneic graft models in mice. GL261 is murine cell line which seems to be immunologically tolerated when implanted in C57BL/6 mice, and this model had been used in some immunological models with some success [17]. Similar to human tumors, GL261 cells have a relatively high fraction of CD133^+ ^glioma cells [18], which are a candidate for the "brain tumor stem cell [18-20]." This cell population has been shown to be relatively non-immunogenic [21], and thus these tumors may model the human condition fairly reliably [21]. The intact T-cell responses in these immunocompetent mice make this model an improvement over xenograft models for studying immunotherapy. The much broader range of reagents, and the much smaller size of mice make testing therapies in mice much easier than in rats, thus giving GL261 model a logistical advantage over other grafting models. Regardless, the implantation methods all suffer from the necessity to introduce foreign tissue into mice to create brain tumors, which likely will always have some immunologic effects.

### Gene Targeted Methods

Mutational analyses of tissue from human brain tumors have revealed that various histopathological categories for primary CNS neoplasia generally result from a limited number of mutation patterns. Recently, transgenic technology has allowed investigators to alter the function of specific genes of interest and thus exploit defined genetic lesions to produce more biologically correct models of CNS cancers that result from activation and/or inactivation of endogenous genes in rodent genomes. A brief summary of presently described models can be found in table 1.

**Table 1:** A summary of existing animal models of brain tumors

	**Tumorigenesis Method**	**Technique**	**Tumor**	**Animal**	**Ref**
Implantation	9 L Gliosarcoma	Syngeneic Graft	GS	Rat	[17]
	C6	Syngeneic Graft	GBM	Rat	[2]
	T9	Syngeneic Graft	GS	Rat	[4]
	RG2	Syngeneic Graft	GBM	Rat	[4]
	F98	Syngeneic Graft	GBM	Rat	[4]
	RT-2	Syngeneic Graft	GBM	Rat	[4]
	CNS-1	Syngeneic Graft	GBM	Rat	[18]
	GL261	Syngeneic Graft	GBM	Mouse	[23]
	Human Tumor Cells (U87, U251)	Xenograft	GBM	Mouse	[5]
Genetic	p53 +/-, NF-1 +/-	Germline mutations	Astro	Mouse	[24]
	GFAP- p53 +/-, NF-1 +/-	Conditional KO	Astro	Mouse	[78]
	GFAP- p53 +/-, NF-1 +/-, PTEN-/-	Conditional KO	Astro	Mouse	[78]
	GFAP- p53 +/-, PTEN-/-	Conditional KO	Astro	Mouse	[87]
	INK4a/ARF -/-, PDGF Overexpression	Germline mutation, RCAS	Astro	Mouse	[47]
	INK4a/ARF -/-, EGF-R overexpression	Germline mutation, RCAS	Astro	Mouse	[48]
	INK4a/ARF -/-, Ras, Akt overexpression	Germline mutation, RCAS	Astro	Mouse	[49]
	Ras, Akt overexpression	RCAS	Astro	Mouse	[80]
	Ras, Akt overexpression, PTEN -/-	RCAS, Conditional KO	Astro	Mouse	[80]
	GFAP-V^12 ^Ras, EGFRvIII	Astrocyte targeted mutation, Adenovirus	Astro	Mouse	[77]
	GFAP-V^12 ^Ras, PTEN -/-	Astrocyte targeted mutation, Germline mutation	Astro	Mouse	[56]
	RAS, EGF-R targeted overexpression	Astrocyte targeted mutations	Astro	Mouse	[73]
	PDGF-B overexpression	MMLV retrovirus	ODG	Mouse	[75]
	PDGF-B overexpression	RCAS	ODG	Mouse	[76]
	Rb inactivation, PTEN -/-	GFAP-Cre targeted conditional KO	ODG		[82]
	INK4a/ARF -/-, PDGF overexp., PTEN -/-	Germline mutation, RCAS, Conditional KO	ODG	Mouse	[88]
	P53 +/-, S100β promoter driven-v-erbB	Germline mutation, Oligodendrocyte mutation	ODG	Mouse	[26]
	INK4a-ARF +/-, S100β promoter v-erbB	Germline mutation, Oligodendrocyte mutation	ODG	Mouse	[26]
	p53 +/-, EGF-R overexpression	Germline mutation, Oligodendrocyte mutation	ODG	Mouse	[48]
	Ptc +/-	Germline mutation or Conditional KO	MB	Mouse	[25]
	Ptc +/-, p53 -/-	Germline mutations	MB	Mouse	[25]
	Shh, n-Myc	RCAS	MB	Mouse	[89]
	Rb +/-, p53 +/-	GFAP-conditional KO	MB	Mouse	[84]
	BRCA2 -/-, p53 +/-	Nestin-conditional KO	MB	Mouse	[86]
	Xrcc4 -/-, p53 -/-	Nestin-conditional KO	MB	Mouse	[81]
	SmoM2	GFAP-conditional KO	MB	Mouse	[79]

(abbreviations (GS-Gliosarcoma, GBM-glioblastoma multiforme, Astro-astrocytoma, ODG-oligodendroglioma, MB-Medulloblastoma, KO-knockout)

While to the genetically modified mouse models are intended to more faithfully recapitulate human brain cancer in animals, little attention has been directed toward the potential flaws in the transgenic paradigm. Many of the genetic mutations required to produce a de novo murine brain tumor, simultaneously interfere with genes involved in a variety of critical immunologic functions. Specific to the current discussion of the immune system, is the observation that processes such as lymphopoesis, the clonal expansion of activated lymphocytes, and the ability of leukocytes to respond to cytokines, rely on the proper functioning of the genes that have been modified in developing transgenic mouse models. This is especially problematic for approaches that involve inducing gliomagenesis by mutating the germ line, and in so doing produce an immunologically flawed paradigm with limited value for pre-clinical testing immunotherapies.

#### p53

The tumor suppressor p53 is a critical regulator of DNA repair, cell cycle regulation, and apoptosis, and is frequently mutated in human cancers, including a significant fraction of secondary GBM. A large number of currently described murine models utilize genetic inactivation of p53 to produce brain tumors. In general, such inhibition is achieved via either germ line p53 deletions, or by functional p53 inhibition utilizing transforming viral proteins.

The germ line approach has been utilized to produce a variety of CNS tumors in mice. For example, Reilly and colleagues found that GBM like lesions developed spontaneously in mice heterozygously deficient in both p53 and the neurofibromatosis-1 gene (nf1) [22]. Wetmore and colleagues reported that medulloblastoma development was accelerated in susceptible Ptc +/- mice by crossing them with p53 -/- homozygotes [23]. Additionally, Weiss and colleagues described a model of oligodendroglioma produced by crossing p53 +/- mice with mice which specifically overexpress EGF-R in oligodendrocytes [24].

Given its central regulatory role in multiple cell processes, it is not surprising that germ line loss of p53 has immunological consequence. Most striking is the very high incidence of spontaneous lymphoma formation in both p53 +/- and p53 -/- mice, consistent with their Li Fraumeni-like genotype [25]. This is likely due to the key role p53 plays in lymphocyte differentiation, as it mediates an important checkpoint in early thymocyte development causing arrest at the CD4-CD8 double negative stage [26,27], regulates the proliferation of pre-B-cells [28], and alters the patterns of expression of Fas on both precursor and mature lymphocytes [29]. Additionally, p53-deficient mice demonstrate impaired B-cell maturation and reduced immunoglobulin deposition in tumors, more rapid aging of the immune system, accumulation of memory T-cells [30], and significantly greater expression of cytokines such as IL-4, IL-6, IL-10, IFN-α [30], osteopontin, and growth/differentiation factor-15 (GDF-15) [31]. Paradoxically, loss of p53 also causes a number of proinflammatory changes at the cellular and organismal level [32]. As well, a large number of immunologically important molecules such as macrophage migration inhibitory factor (MIF) [33], IL-6 [34], IFN-α [35], IFN-β [36], and NF-κB [37] are known to mediate at least some of their effects through p53. In addition, thymocytes from p53 deficient mice demonstrate increased resistance to radiation induced apoptosis [38,39], and p53 deficiency alters autoantibody levels in models of autoimmunity [40] as well as reduces mast cell susceptibility to IFN-γ induced apoptosis [41]. Given these observations, it seems likely that the pan-suppression of p53 activity introduced by the use of germ line p53 inactivation alters immune system function in a number of significant ways in these animals, limiting the use of these models for evaluating the effect of anti-tumor immunotherapies. Other research groups have shown that CNS tumors can be produced by cell-targeted introduction of viral antigens that suppress p53 activity. Probably the most immunologically correct method for accomplishing this are conditional knockout methods (described below), although a number of other methods exist. For example, Chiu and colleagues demonstrated that mice possessing an SV40 T-antigen transgene (which functionally inactivates Rb and p53), driven by the brain specific FGF-1B promoter, develop poorly differentiated tumors of the medulla and 4^th ^ventricle which closely resemble primitive neuroectodermal tumors (PNET) [42]. An alternate approach, described by Krynska and colleagues, also produced PNET-like tumors by creating mice transgenic for the early region of the CY variant of the JC virus, which encodes a T-antigen that inhibits both p53 and Rb. To some extent, these models represent an improvement over germ line based models because they limit the effects of p53 inhibition to specific cells. However the introduction of viral antigens expressed in tumor cells, has great potential to alter the interaction of the immune systems with these tumors [43].

#### INK4a/ARF

The tumor suppressor locus INK4a/ARF encodes two tumor suppressor genes: p16^INK4a^, which prevents Rb phosphorylation by binding CDK4; and p14/p19^ARF^, which prevents p53 degradation via MDM2 inhibition [44]. Loss of function mutation of one or both gene products encoded by INK4a/ARF is a common mutation in human cancer, including glioma [44], and accordingly numerous investigators have utilized INK4a/ARF silencing mutations to create CNS neoplasms in mice. Dai and colleagues demonstrated that oligodenrogliomas and oligoastrocytomas could be produced in INK4a/ARF -/- mice by forcing glial precursor cells to overexpress PDGF, using the RCAS system [45], which involves delivery of oncogene-encoding viral vectors to cells that have been engineered to express receptor for RCAS virus. Using the same system, this group has described the production high grade gliomas by combining INK4a/ARF deletion with astrocyte specific overexpression of EGFR [46], or Ras and Akt [47]. The immunologic significance of a tumor expressing RCAS antigens has yet to be addressed, and because all of these models share the common trait of utilizing germline INK4a/Arf deletion to promote glial neoplasms, there are undoubtedly additional immunologic consequences of these models that would not be encountered in patients where INK4a/Arf inactivation was limited to tumor cells only. For example, in a manner similar to p53 deficient mice, ARF -/- mice are known to spontaneously develop lymphomas in the absence of other mutations [48]. This is not surprising, given the important role these genes play in cell cycle regulation in developing thymocytes [49,50]. As well, p14/p19^ARF ^plays a role in suppressing the respiratory burst in neutrophils [51,52].

##### Phosphatase and Tensin Homolog *(PTEN)*

PTEN is a tumor suppressor gene which inhibits cell proliferation and growth via suppression of the PI3-kinase signaling pathway [53]. Loss of function mutations of PTEN have been observed in approximately 50% of de novo GBM patients [54]. One significance of this observation was revealed by Xiao and colleagues who reported that crossbreeding PTEN +/- mice with a strain containing a GFAP driven truncated SV40 T antigen resulted in Rb, p107, and p130 (but not p53) inhibition, and significantly accelerated the development of GBM in the double transgenic progeny [54]. Here again, the use of PTEN germ line mutations is problematic for immunological studies using this model. Similar to other tumor suppressor genes, PTEN plays a critical role in lymphocyte development, serving to eliminate T-cells that do not produce an effective TCR re-arrangement [55]. Not surprisingly, PTEN +/- mice have been demonstrated to frequently develop T-cell lymphomas [55,56], as well as diffuse lymphoid hyperplasia [57,58]. In addition, PTEN appears to regulate leukocyte chemotaxis at a variety of levels, including regulation of CXCR4 expression [59], which directs actin polymerization during chemotaxis [60]. It is unclear whether or not T cells from these transgenic animals are fully functional.

##### Epidermal Growth Factor Receptor (EGF-R)

EGF-R is a member of the ErbB tyrosine kinase receptor family that is mutated or overexpressed in a variety of human tumors, including approximately 30-50% of primary glioblastoma multiforme [61] and in roughly half of oligodendrogliomas [62]. In addition to its role in neoplasia, EGF-R plays a pivotal role as a so called "master switch" which modulates of a broad variety of immunological functions [63]. For example, EGF-R activation appears to sensitize neutrophils to the effects of TNF-α, leading to increased expression of the adhesion molecule CD-11b, increased IL-8 production, and improved respiratory burst by these "EGF-R primed" cells [64]. EGF-R mediates chemotaxis in peripheral blood monocytes and monocyte derived macrophages [65], and is critical for the response of myeloid lineage cells to colony stimulating factors [66]. EGF-R activation stimulates release of IL-8 from cultured bronchial epithelial cells [67], and is hypothesized to play a critical role in the pathogenesis of inflammatory lung diseases such as panbronchitis and asthma [67,68]. EGF-R down-regulates CCL2, CCL5, and CXCL10, and increases CXCL8 in keratinocytes which likely propagates the pro-inflammatory state seen in autoimmune skin disorders [69]. Finally, EGF-R is required for cytokine dependent production of nitric oxide by the pulmonary vasculature [70].

To date, there have been several reports demonstrating the use of EGF-R overexpression to produce either oligodenroglioma or astrocytoma-like tumors in mice. Holland and colleagues reported that virus expressing EGFRvIII (a common mutant form of EGFR), and used to infect INK4a-ARF null astrocytes or glial precursors (via the RCAS system described above), produce gliomas in transgenic mice [46]. Weiss and colleagues demonstrated that oligodendrogliomas reliably occur in mice doubly transgenic for an S100β promoter driven-v-erbB (a transforming EGF-R allele), and either INK4a-ARF +/- or P53 +/- heterozygosity [24]. Ding and colleagues have reported the development of oligodendrogliomas and mixed oligoastrocytomas in mice carrying RAS and EGF-R transgenes driven by GFAP promoters [71]. In all three models, the use of glial specific promoters likely minimize the systemic effects of EGF-R overexpression on immune function. However the dependence of EGF-R models on the use of cross breeding with germ line mutants, likely introduces its own set of immunobiological consequences, as discussed earlier.

### Platelet Derived Growth Factor (PDGF)

PDGF is a growth factor that is expressed in many normal tissues and mediates a variety of effects on cell growth and differentiation via induced dimerization-activation of its corresponding tyrosine kinase receptor, PDGF-R. Overexpression of both the PDGF isoform, PDGF-B, and the receptor PDGF-R frequently occur in gliomas, suggesting the potential role of a malfunctioning autocrine signaling loop in the pathogenesis of some of these tumors [72]. Existing PDGF based models typically utilize approaches that limit ligand overexpression to the peritumoral region, or at least the CNS. For example, Hesselager and colleagues found that using a MMLV retroviral construct to drive PDGF-B expression it was possible to induce gliomagenesis in neonatal mice brains, and in the absence of other mutations (though additional relevant mutations appeared to accelerate tumor growth) [73]. Dai and colleagues have demonstrated that oligodendrogliomas could be produced solely by introduction of PDGF-B overexpression using the RCAS system, and that this process was accelerated by the addition of INK4a/ARF p53 germline mutations [74].

While both models have the desirable feature of causing gliomagenesis with minimal effects to the host immune system, little attention has been directed towards analyzing the effects of overexpression of a soluble leukocyte chemoattractant. It is important to know whether PDGF-driven tumors secrete similar levels of PDGF as their naturally occurring counterparts, and what effect PDGF overexpression has on local intratumoral inflammatory responses.

## Tissue Targeting with Conditional Knockouts

Tissue specific overexpression of putative oncogenes of interest, using methods which link the gene of interest to a glial specific promoter such as GFAP, S100β, or Nestin, provides an appealing approach towards the creation of spontaneously occurring brain tumors in animals that lack the pan-immune dysfunctions seen in many germline knockout animals [75]. Tissue targeted models involving deletion of tumor suppressor genes is more difficult, which is why most models to described to date have utilized germline knockouts to reduce tumor suppressor gene function. Conditional knockout models represent a promising new attempt to eliminate tumor suppressor function in a cell specific manner [76,77]. For brain tumors, this involves GFAP or Nestin driven expression of the bacteriophage protein Cre, which removes sections of DNA between E. Coli specific DNA sequences known as loxP domains [76]. By co-introducing Cre driven on tissue specific promoters, and the tumor suppressor gene of interest flanked by loxP regions, it is possible to knock out tumor suppressor genes of interest in the cell type of choice [76]. These techniques have recently been utilized to create of variety of transgenic brain tumor models using targeted conditional knockouts of p53 [78,79], PTEN [80], Ptc [81], and Rb [82]. Frequently, conditional knockouts used in combination with oncogenes overexpressed on tissue specific promoters or introduced using viral vectors can create a localized tumor genetically similar to human cancer in an immune competent animal [74]. While these and other similar models [83-85] certainly represent an improvement over germline mutation based models [86], the constitutive expression of a bacteriophage protein in the cell of interest, raises some concern regarding the immunogenicity of the tumor cells created in this manner, and deserves future attention.

## Conclusion

Animal models represent essential tools understanding complex molecular and cellular interactions occurring in brain tumors, and for the evaluation of potential therapies. Rodents do not typically develop CNS neoplasms spontaneously, and it is important that we understand the physiologic changes induced by the methods used to create thse tumors, and adjust our interpretation of results obtained with these models accordingly. Significant improvements have been made over the last decade to induce gliomas using tissue targeted conditional deletions and cell specific oncogene overexpression. While existing models may represent improvements over chemically induced rodent syngeneic models, the immunologic effects of these methods are not entirely understood, and deserve more investigation.

## Conflicting interests

The authors declare that they have no competing interests.

## Authors' contributions

All authors read and approved this manuscript. MS provided the manuscript idea, and prepared the manuscript. IY also provided the manuscript idea, and helped prepared the manuscript. AK, MR, and SF helped with literature searches and manuscript preparation and editing. DJ helped edit the manuscript and contributed insight from his experience in the field. AP helped generate the manuscript idea and contributed significantly to the manuscript's final form.

## References

[B1] Stupp R, Mason WP, Bent MJ van den, Weller M, Fisher B, Taphoorn MJ, Belanger K, Brandes AA, Marosi C, Bogdahn U (2005). Radiotherapy plus concomitant and adjuvant temozolomide for glioblastoma. N Engl J Med.

[B2] Parsa AT, Chakrabarti I, Hurley PT, Chi JH, Hall JS, Kaiser MG, Bruce JN (2000). Limitations of the C6/Wistar rat intracerebral glioma model: implications for evaluating immunotherapy. Neurosurgery.

[B3] Paek SH, Chung HT, Jeong SS, Park CK, Kim CY, Kim JE, Kim DG, Jung HW (2005). Hearing preservation after gamma knife stereotactic radiosurgery of vestibular schwannoma. Cancer.

[B4] Barth RF (1998). Rat brain tumor models in experimental neuro-oncology: the 9L, C6, T9, F98, RG2 (D74), RT-2 and CNS-1 gliomas. J Neurooncol.

[B5] Delgado C, Hoa N, Callahan LL, Schiltz PM, Jahroudi RA, Zhang JG, Wepsic HT, Jadus MR (2007). Generation of human innate immune responses towards membrane macrophage colony stimulating factor (mM-CSF) expressing U251 glioma cells within immunodeficient (NIH-nu/beige/xid) mice. Cytokine.

[B6] Kim HM, Kang JS, Lim J, Kim JY, Kim YJ, Lee SJ, Song S, Hong JT, Kim Y, Han SB (2009). Antitumor activity of cytokine-induced killer cells in nude mouse xenograft model. Arch Pharm Res.

[B7] Wang P, Yu JP, Gao SY, An XM, Ren XB, Wang XG, Li WL (2008). Experimental study on the treatment of intracerebral glioma xenograft with human cytokine-induced killer cells. Cell Immunol.

[B8] Anderson RC, Elder JB, Brown MD, Mandigo CE, Parsa AT, Kim PD, Senatus P, Anderson DE, Bruce JN (2002). Changes in the immunologic phenotype of human malignant glioma cells after passaging in vitro. Clinical Immunology.

[B9] Li A, Walling J, Kotliarov Y, Center A, Steed ME, Ahn SJ, Rosenblum M, Mikkelsen T, Zenklusen JC, Fine HA (2008). Genomic changes and gene expression profiles reveal that established glioma cell lines are poorly representative of primary human gliomas. Mol Cancer Res.

[B10] Barker M, Hoshino T, Gurcay O, Wilson CB, Nielsen SL, Downie R, Eliason J (1973). Development of an animal brain tumor model and its response to therapy with 1,3-bis(2-chloroethyl)-1-nitrosourea. Cancer Research.

[B11] Kruse CA, Lillehei KO, Mitchell DH, Kleinschmidt-DeMasters B, Bellgrau D (1990). Analysis of interleukin 2 and various effector cell populations in adoptive immunotherapy of 9L rat gliosarcoma: allogeneic cytotoxic T lymphocytes prevent tumor take. Proceedings of the National Academy of Sciences of the United States of America.

[B12] Kruse CA, Mitchell DH, Kleinschmidt-DeMasters BK, Bellgrau D, Eule JM, Parra JR, Kong Q, Lillehei KO (1993). Systemic chemotherapy combined with local adoptive immunotherapy cures rats bearing 9L gliosarcoma. Journal of Neuro-Oncology.

[B13] Kruse CA, Schiltz PM, Bellgrau D, Kong Q, Kleinschmidt-DeMasters BK (1994). Intracranial administrations of single or multiple source allogeneic cytotoxic T lymphocytes: chronic therapy for primary brain tumors. Journal of Neuro-Oncology.

[B14] Kruse CA, Molleston MC, Parks EP, Schiltz PM, Kleinschmidt-DeMasters BK, Hickey WF (1994). A rat glioma model, CNS-1, with invasive characteristics similar to those of human gliomas: a comparison to 9L gliosarcoma. Journal of Neuro-Oncology.

[B15] Kruse CA, Kong Q, Schiltz PM, Kleinschmidt-DeMasters BK (1994). Migration of activated lymphocytes when adoptively transferred into cannulated rat brain. Journal of Neuroimmunology.

[B16] Blume MRWCaVD, Sane K ISaLD (1974). Immune response to a transplantable intracerebral glioma in rats. Recent Progress in Neurologic Surgery.

[B17] Glick RP, Lichtor T, de Zoeten E, Deshmukh P, Cohen EP (1999). Prolongation of survival of mice with glioma treated with semiallogeneic fibroblasts secreting interleukin-2. Neurosurgery.

[B18] Wu A, Oh S, Wiesner SM, Ericson K, Chen L, Hall WA, Champoux PE, Low WC, Ohlfest JR (2008). Persistence of CD133+ cells in human and mouse glioma cell lines: detailed characterization of GL261 glioma cells with cancer stem cell-like properties. Stem Cells Dev.

[B19] Zhang M, Song T, Yang L, Chen R, Wu L, Yang Z, Fang J (2008). Nestin and CD133: valuable stem cell-specific markers for determining clinical outcome of glioma patients. J Exp Clin Cancer Res.

[B20] Coskun V, Wu H, Blanchi B, Tsao S, Kim K, Zhao J, Biancotti JC, Hutnick L, Krueger RC, Fan G (2008). CD133+ neural stem cells in the ependyma of mammalian postnatal forebrain. Proc Natl Acad Sci USA.

[B21] Abdouh M, Facchino S, Chatoo W, Balasingam V, Ferreira J, Bernier G (2009). BMI1 sustains human glioblastoma multiforme stem cell renewal. J Neurosci.

[B22] Reilly KM, Loisel DA, Bronson RT, McLaughlin ME, Jacks T (2000). Nf1;Trp53 mutant mice develop glioblastoma with evidence of strain-specific effects. Nature Genetics.

[B23] Wetmore C, Eberhart DE, Curran T (2001). Loss of p53 but not ARF accelerates medulloblastoma in mice heterozygous for patched. Cancer Research.

[B24] Weiss WA, Burns MJ, Hackett C, Aldape K, Hill JR, Kuriyama H, Kuriyama N, Milshteyn N, Roberts T, Wendland MF (2003). Genetic determinants of malignancy in a mouse model for oligodendroglioma. Cancer Research.

[B25] Donehower LA (1996). The p53-deficient mouse: a model for basic and applied cancer studies. Seminars in Cancer Biology.

[B26] Okazuka K, Wakabayashi Y, Kashihara M, Inoue J, Sato T, Yokoyama M, Aizawa S, Aizawa Y, Mishima Y, Kominami R (2005). p53 prevents maturation of T cell development to the immature CD4-CD8+ stage in Bcl11b-/- mice. Biochemical & Biophysical Research Communications.

[B27] Sulic S, Panic L, Barkic M, Mercep M, Uzelac M, Volarevic S (2005). Inactivation of S6 ribosomal protein gene in T lymphocytes activates a p53-dependent checkpoint response. Genes Dev.

[B28] Hu L, Aizawa S, Tokuhisa T (1995). p53 Controls Proliferation of Early B Lineage Cells by a P21 (WAF1/Cip1)-Independent Pathway. Biochemical and Biophysical Research Communications.

[B29] Li T, Ramirez K, Palacios R (1996). Distinct patterns of Fas cell surface expression during development of T- or B-lymphocyte lineages in normal, scid, and mutant mice lacking or overexpressing p53, bcl-2, or rag-2 genes. Cell Growth & Differentiation.

[B30] Ohkusu-Tsukada K, Tsukada T, Isobe K-i (1999). Accelerated Development and Aging of the Immune System in p53-Deficient Mice. J Immunol.

[B31] Zimmers TA, Jin X, Hsiao EC, McGrath SA, Esquela AF, Koniaris LG (2005). Growth Differentiation Factor-15/Macrophage Inhibitory Cytokine-1 Induction After Kidney and Lung Injury. Shock.

[B32] Komarova EA, Krivokrysenko V, Wang K, Neznanov N, Chernov MV, Komarov PG, Brennan M-L, Golovkina TV, Rokhlin O, Kuprash DV (2005). p53 is a suppressor of inflammatory response in mice. FASEB J.

[B33] Morand EF (2005). New therapeutic target in inflammatory disease: macrophage migration inhibitory factor. Internal Medicine Journal.

[B34] Hodge DR, Peng B, Cherry JC, Hurt EM, Fox SD, Kelley JA, Munroe DJ, Farrar WL (2005). Interleukin 6 Supports the Maintenance of p53 Tumor Suppressor Gene Promoter Methylation. Cancer Res.

[B35] Porta C, Hadj-Slimane R, Nejmeddine M, Pampin M, Tovey MG, Espert L, Alvarez S, Chelbi-Alix MK (2004). Interferons [alpha] and [gamma] induce p53-dependent and p53-independent apoptosis, respectively.

[B36] Shin-Ya M, Hirai H, Satoh E, Kishida T, Asada H, Aoki F, Tsukamoto M, Imanishi J, Mazda O (2005). Intracellular interferon triggers Jak/Stat signaling cascade and induces p53-dependent antiviral protection. Biochemical and Biophysical Research Communications.

[B37] Gomez J, Garcia-Domingo D, Martinez AC, Rebollo A (1997). Role of NF-kappaB in the control of apoptotic and proliferative responses in IL-2-responsive T cells. Frontiers in Bioscience.

[B38] Maas K, Westfall M, Pietenpol J, Olsen NJ, Aune T (2005). Reduced p53 in peripheral blood mononuclear cells from patients with rheumatoid arthritis is associated with loss of radiation-induced apoptosis. Arthritis & Rheumatism.

[B39] Lowe SW, Schmitt EM, Smith SW, Osborne BA, Jacks T (1993). p53 is required for radiation-induced apoptosis in mouse thymocytes[see comment]. Nature.

[B40] Kuan AP, Cohen PL (2005). p53 is required for spontaneous autoantibody production in B6/lpr lupus mice. European Journal of Immunology.

[B41] Mann-Chandler MN, Kashyap M, Wright HV, Norozian F, Barnstein BO, Gingras S, Parganas E, Ryan JJ (2005). IFN-{gamma} Induces Apoptosis in Developing Mast Cells. J Immunol.

[B42] Chiu IM, Touhalisky K, Liu Y, Yates A, Frostholm A (2000). Tumorigenesis in transgenic mice in which the SV40 T antigen is driven by the brain-specific FGF1 promoter. Oncogene.

[B43] Krynska B, Otte J, Franks R, Khalili K, Croul S (1999). Human ubiquitous JCV(CY) T-antigen gene induces brain tumors in experimental animals. Oncogene.

[B44] Ivanchuk SM, Mondal S, Dirks PB, Rutka JT (2001). The INK4A/ARF Locus: Role in Cell Cycle Control and Apoptosis and Implications for Glioma Growth. Journal of Neuro-Oncology.

[B45] Dai C, Krantz SB (2001). Increased expression of the INK4a/ARF locus in polycythemia vera. Blood.

[B46] Holland EC, Hively WP, DePinho RA, Varmus HE (1998). A constitutively active epidermal growth factor receptor cooperates with disruption of G1 cell-cycle arrest pathways to induce glioma-like lesions in mice. Genes & Development.

[B47] Uhrbom L, Dai C, Celestino JC, Rosenblum MK, Fuller GN, Holland EC (2002). Ink4a-Arf loss cooperates with KRas activation in astrocytes and neural progenitors to generate glioblastomas of various morphologies depending on activated Akt. Cancer Research.

[B48] Kamijo T, Zindy F, Roussel MF, Quelle DE, Downing JR, Ashmun RA, Grosveld G, Sherr CJ (1997). Tumor Suppression at the Mouse INK4a Locus Mediated by the Alternative Reading Frame Product p19 ARF. Cell.

[B49] Migliaccio M, Raj K, Menzel O, Rufer N (2005). Mechanisms that limit the in vitro proliferative potential of human CD8+ T lymphocytes. Journal of Immunology.

[B50] Scheuring UJ, Sabzevari H, Theofilopoulos AN (2002). Proliferative arrest and cell cycle regulation in CD8(+)CD28(-) versus CD8(+)CD28(+) T cells. Human Immunology.

[B51] Wang JP, Chang LC, Hsu MF, Lin CN (2003). The blockade of formyl peptide-induced respiratory burst by 2',5'-dihydroxy-2-furfurylchalcone involves phospholipase D signaling in neutrophils. Naunyn-Schmiedebergs Archives of Pharmacology.

[B52] Wang JP, Chang LC, Hsu MF, Chen SC, Kuo SC (2002). Inhibition of formyl-methionyl-leucyl-phenylalanine-stimulated respiratory burst by cirsimaritin involves inhibition of phospholipase D signaling in rat neutrophils. Naunyn-Schmiedebergs Archives of Pharmacology.

[B53] Leslie NR, Downes CP (2004). PTEN function: how normal cells control it and tumour cells lose it. Biochemical Journal.

[B54] Xiao A, Wu H, Pandolfi PP, Louis DN, Van Dyke T (2002). Astrocyte inactivation of the pRb pathway predisposes mice to malignant astrocytoma development that is accelerated by PTEN mutation. Cancer Cell.

[B55] Hagenbeek TJ, Naspetti M, Malergue F, Garcon F, Nunes JA, Cleutjens KB, Trapman J, Krimpenfort P, Spits H (2004). The Loss of PTEN Allows TCR {alpha}{beta} Lineage Thymocytes to Bypass IL-7 and Pre-TCR-mediated Signaling. J Exp Med.

[B56] Suzuki A, de la Pompa JL, Stambolic V, Elia AJ, Sasaki T, del Barco Barrantes I, Ho A, Wakeham A, Itie A, Khoo W (1998). High cancer susceptibility and embryonic lethality associated with mutation of the PTEN tumor suppressor gene in mice. Current Biology.

[B57] Stambolic V, Tsao MS, Macpherson D, Suzuki A, Chapman WB, Mak TW (2000). High incidence of breast and endometrial neoplasia resembling human Cowden syndrome in pten +/- mice. Cancer Research.

[B58] Luo J, Sobkiw CL, Logsdon NM, Watt JM, Signoretti S, O'Connell F, Shin E, Shim Y, Pao L, Neel BG (2005). Modulation of epithelial neoplasia and lymphoid hyperplasia in PTEN +/- mice by the p85 regulatory subunits of phosphoinositide 3-kinase. PNAS.

[B59] Gao P, Wange RL, Zhang N, Oppenheim JJ, Howard OMZ (2005). Negative regulation of CXCR4-mediated chemotaxis by the lipid phosphatase activity of tumor suppressor PTEN. Blood.

[B60] Lacalle RA, Gomez-Mouton C, Barber DF, Jimenez-Baranda S, Mira E, Martinez AC, Carrera AC, Manes S (2004). PTEN regulates motility but not directionality during leukocyte chemotaxis. Journal of Cell Science.

[B61] Libermann TA, Nusbaum HR, Razon N, Kris R, Lax I, Soreq H, Whittle N, Waterfield MD, Ullrich A, Schlessinger J (1985). Amplification, enhanced expression and possible rearrangement of EGF receptor gene in primary human brain tumours of glial origin. Nature.

[B62] Reifenberger J, Reifenberger G, Ichimura K, Schmidt EE, Wechsler W, Collins VP (1996). Epidermal growth factor receptor expression in oligodendroglial tumors. American Journal of Pathology.

[B63] Yan S-F, Fujita T, Lu J, Okada K, Shan Zou Y, Mackman N, Pinsky DJ, Stern DM (2000). Egr-1, a master switch coordinating upregulation of divergent gene families underlying ischemic stress.

[B64] Lewkowicz P, Tchorzewski H, Dytnerska K, Banasik M, Lewkowicz N (2005). Epidermal growth factor enhances TNF-alpha-induced priming of human neutrophils. Immunology Letters.

[B65] Lamb DJ, Modjtahedi H, Plant NJ, Ferns GA (2004). EGF mediates monocyte chemotaxis and macrophage proliferation and EGF receptor is expressed in atherosclerotic plaques. Atherosclerosis.

[B66] Watanabe S, Yoshimura A, Inui K, Yokota N, Liu Y, Sugenoya Y, Morita H, Ideura T (2001). Acquisition of the monocyte/macrophage phenotype in human mesangial cells. Journal of Laboratory & Clinical Medicine.

[B67] Hamilton LM, Torres-Lozano C, Puddicombe SM, Richter A, Kimber I, Dearman RJ, Vrugt B, Aalbers R, Holgate ST, Djukanovic R (2003). The role of the epidermal growth factor receptor in sustaining neutrophil inflammation in severe asthma. Clinical & Experimental Allergy.

[B68] Kim JH, Jung KH, Han JH, Shim JJ, In KH, Kang KH, Yoo SH (2004). Relation of epidermal growth factor receptor expression to mucus hypersecretion in diffuse panbronchiolitis. Chest.

[B69] Mascia F, Mariani V, Girolomoni G, Pastore S (2003). Blockade of the EGF receptor induces a deranged chemokine expression in keratinocytes leading to enhanced skin inflammation. American Journal of Pathology.

[B70] Nelin LD, Chicoine LG, Reber KM, English BK, Young TL, Liu Y (2005). Cytokine-induced endothelial arginase expression is dependent on epidermal growth factor receptor. American Journal of Respiratory Cell & Molecular Biology.

[B71] Ding H, Shannon P, Lau N, Wu X, Roncari L, Baldwin RL, Takebayashi H, Nagy A, Gutmann DH, Guha A (2003). Oligodendrogliomas result from the expression of an activated mutant epidermal growth factor receptor in a RAS transgenic mouse astrocytoma model. Cancer Research.

[B72] Hermanson M, Funa K, Hartman M, Claesson-Welsh L, Heldin C, Westermark B, Nister M (1992). Platelet-derived growth factor and its receptors in human glioma tissue: expression of messenger RNA and protein suggests the presence of autocrine and paracrine loops. Cancer Res.

[B73] Hesselager G, Uhrbom L, Westermark B, Nister M (2003). Complementary Effects of Platelet-derived Growth Factor Autocrine Stimulation and p53 or Ink4a-Arf Deletion in a Mouse Glioma Model. Cancer Res.

[B74] Dai C, Celestino JC, Okada Y, Louis DN, Fuller GN, Holland EC (2001). PDGF autocrine stimulation dedifferentiates cultured astrocytes and induces oligodendrogliomas and oligoastrocytomas from neural progenitors and astrocytes in vivo. Genes Dev.

[B75] Wei Q, Clarke L, Scheidenhelm DK, Qian B, Tong A, Sabha N, Karim Z, Bock NA, Reti R, Swoboda R (2006). High-grade glioma formation results from postnatal pten loss or mutant epidermal growth factor receptor expression in a transgenic mouse glioma model. Cancer Res.

[B76] Kwon CH, Zhao D, Chen J, Alcantara S, Li Y, Burns DK, Mason RP, Lee EY, Wu H, Parada LF (2008). Pten haploinsufficiency accelerates formation of high-grade astrocytomas. Cancer Res.

[B77] Schuller U, Heine VM, Mao J, Kho AT, Dillon AK, Han YG, Huillard E, Sun T, Ligon AH, Qian Y (2008). Acquisition of granule neuron precursor identity is a critical determinant of progenitor cell competence to form Shh-induced medulloblastoma. Cancer Cell.

[B78] Hu X, Pandolfi PP, Li Y, Koutcher JA, Rosenblum M, Holland EC (2005). mTOR promotes survival and astrocytic characteristics induced by Pten/AKT signaling in glioblastoma. Neoplasia.

[B79] Yan CT, Kaushal D, Murphy M, Zhang Y, Datta A, Chen C, Monroe B, Mostoslavsky G, Coakley K, Gao Y (2006). XRCC4 suppresses medulloblastomas with recurrent translocations in p53-deficient mice. Proc Natl Acad Sci USA.

[B80] Xiao A, Yin C, Yang C, Di Cristofano A, Pandolfi PP, Van Dyke T (2005). Somatic induction of Pten loss in a preclinical astrocytoma model reveals major roles in disease progression and avenues for target discovery and validation. Cancer Res.

[B81] Yang ZJ, Ellis T, Markant SL, Read TA, Kessler JD, Bourboulas M, Schuller U, Machold R, Fishell G, Rowitch DH (2008). Medulloblastoma can be initiated by deletion of Patched in lineage-restricted progenitors or stem cells. Cancer Cell.

[B82] Marino S, Vooijs M, Gulden H van Der, Jonkers J, Berns A (2000). Induction of medulloblastomas in p53-null mutant mice by somatic inactivation of Rb in the external granular layer cells of the cerebellum. Genes Dev.

[B83] Frappart PO, Lee Y, Lamont J, McKinnon PJ (2007). BRCA2 is required for neurogenesis and suppression of medulloblastoma. EMBO J.

[B84] Zheng H, Ying H, Yan H, Kimmelman AC, Hiller DJ, Chen AJ, Perry SR, Tonon G, Chu GC, Ding Z (2008). p53 and Pten control neural and glioma stem/progenitor cell renewal and differentiation. Nature.

[B85] Huse JT, Holland EC (2009). Genetically engineered mouse models of brain cancer and the promise of preclinical testing. Brain Pathol.

[B86] Rao G, Pedone CA, Coffin CM, Holland EC, Fults DW (2003). c-Myc enhances sonic hedgehog-induced medulloblastoma formation from nestin-expressing neural progenitors in mice. Neoplasia.

